# SIRT4 enhances the sensitivity of ER‐positive breast cancer to tamoxifen by inhibiting the IL‐6/STAT3 signal pathway

**DOI:** 10.1002/cam4.2557

**Published:** 2019-10-01

**Authors:** Jilin Xing, Ji Li, Lin Fu, Junda Gai, Jingqian Guan, Qingchang Li

**Affiliations:** ^1^ Department of Pathology First Affiliated Hospital and College of Basic Medical Sciences China Medical University Shenyang China

**Keywords:** *CCND1*, *MYC*, *SIRT4*, *STAT3*, tamoxifen

## Abstract

Recent advances in endocrine therapy have improved the prospects for estrogen receptor‐positive breast cancer. Tamoxifen is an effective drug for patients with estrogen receptor‐positive breast cancer, but the development of resistance is common. Therefore, discovering ways to enhance the sensitivity of cancer cells to tamoxifen may help improve breast cancer treatment. We studied the biological role of sirtuin 4 (SIRT4) in tamoxifen‐treated MCF7 and T47D cells. The levels of the MYC proto‐oncogene (MYC) and cyclin D1 (CCND1) were detected by western blotting and quantitative reverse transcription‐polymerase chain reaction in breast cancer cells with SIRT4 overexpression or depletion. Immunofluorescence and western blotting were used to assess the phosphorylation status of signal transducer and activator of transcription 3 (STAT3). SIRT4 overexpression decreased the half maximal inhibitory concentration of tamoxifen in MCF7 and T47D cells, while its depletion increased it. Thus, SIRT4 enhances the sensitivity of breast cancer cells to tamoxifen. Moreover, western blotting revealed decreased STAT3 phosphorylation after SIRT4 transfection. The transcription and translation of *MYC* and *CCND1*, target genes of the STAT3 pathway, were also blocked. Immunofluorescence revealed that pathway activation and nuclear STAT4 translocation were suppressed when SIRT4 was overexpressed. Furthermore, the effects of SIRT4 overexpression or depletion on proliferation could be offset by STAT3 activation or inhibition. Taken together, these results demonstrate that SIRT4 enhances the tamoxifen sensitivity of breast cancer cells by inhibiting the STAT3 signaling pathway. With this knowledge, therapeutic strategies with reduced drug resistance risk may be developed.

## Graphical abstract

We proved for the first time that SIRT4 enhances sensitivity of breast cancer cells to tamoxifen. Western blotting showed that p‐STAT3 levels were reduced after SIRT4 transfection and that transcription and translation of MYC and CCND1 were blocked. Immunofluorescence revealed that STAT3 activation and nuclear translocation were suppressed when SIRT4 was overexpressed.

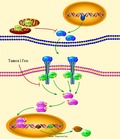

## 1 INTRODUCTION

Sirtuin 4 (SIRT4), a mitochondrial protein, is a member of the highly conserved sirtuin protein family and has ADP‐ribonucleotransferase activity. SIRT4 ADP‐ribosylates glutamate dehydrogenase, which prevents glutamate conversion to *α*‐ketoglutarate, blocking the tricarboxylic acid cycle.^1^ In this way, SIRT4 inhibits glutamine metabolism. Recent studies have shown that *SIRT4* is a tumor suppressor gene in many cancers.^2^, ^3^ However, few studies have examined the roles of SIRT4 in breast cancer, which occurs in mammary gland epithelial tissue and is one of the most common malignant tumors worldwide. It is a serious threat to the health of women, and the majority of cases are estrogen receptor (ER)‐positive.^4^ Tamoxifen, a competitive estradiol antagonist, is the first‐line endocrine therapy for (ER)‐positive breast cancer. Tamoxifen kills breast cancer cells not only by binding to estrogen receptors but also by blocking glutamine uptake, reducing glutathione production.^5^ Given the impact of SIRT4 on glutamine metabolism, we hypothesized that SIRT4 may affect the sensitivity of ER‐positive breast cancer to tamoxifen.

Signal transducer and activator of transcription 3 (STAT3) mediates the expression of a variety of genes in response to cell stimuli and thus plays key roles in many cellular processes, such as cell growth and apoptosis. STAT3 hyperactivation via the phosphorylation of tyrosine 705 (Y705) is common in most human cancers.^6^ In addition, elevated levels of STAT3 Y705 phosphorylation have been observed in tamoxifen‐resistant MCF‐7/TAM cells. In this study, we evaluated whether SIRT4 inhibits p‐STAT3 Y705 in ER‐positive breast cancer cells.

The MYC proto‐oncogene (*MYC*) and cyclin D1 (*CCND1*) are target genes of the STAT3 pathway. *MYC* is upregulated in the tamoxifen‐resistant breast cancer cell line MCF7/TAM, and these cells are more sensitive to tamoxifen after *MYC* knockout. ER‐positive tumors with *CCND1* amplification are not sensitive to tamoxifen.^7^, ^8^

## 2 METHODS

### 2.1 Cell lines and transfection

The MCF7 and T47D breast cancer cell lines were obtained from the Shanghai Cell Bank (Shanghai, China). MCF7 cells were cultured in Dulbecco's modified Eagle's medium, and T47D cells were cultured in Roswell Park Memorial Institute 1640 medium, both supplemented with 10% fetal bovine serum.

The SIRT4 coding sequence was cloned into a pCMV6‐Entry vector (OriGene, Rockville, MD, USA). The STAT3 coding sequence was cloned into a pLEGFP‐N1‐neo vector (Addgene, Cambridge, MA, USA). The breast cancer cells were transfected with Lipofectamine 3000 (Thermo Fisher Scientific, Waltham, MA, USA). The negative control was obtained by transfection with blank vectors. SIRT4 short interfering RNA (siRNA), negative controls, and STAT3 siRNA were purchased from RuiBo (Shanghai, China). Cells were used 48‐72 hours after transfection.

### 2.2 Enzyme‐linked immunosorbent assay (ELISA)

Glutamine levels in the medium were detected using a human glutamine ELISA kit (Lanpai BIO, Shanghai, China). After preparing the Microelisa Stripplate, standard wells and testing sample wells were set. Standard wells received 50 μL of standard; sample wells received 10 μL of test sample and 40 μL of sample diluent. After addition of a horseradish peroxidase (HRP)‐conjugated reagent, the wells were covered with an adhesive strip and incubated for 60 minutes at 37°C. The wells were then aspirated and washed four times, then the plates were inverted and blotted with clean paper towels. Chromogen solutions A and B (50 μL each) were added to each well, gently mixed in the dark, and incubated for 15 minutes at 37°C. Then stop solution (50 μL) was added to each well, and the optical density (OD) values of the wells were read at 450 nm within 15 minutes.

### 2.3 CCK‐8 assay

CCK8 is used to measure relative cell viability and proliferation. Cell suspensions (100 μL; 50 000 cells/mL) were placed in 96‐well plates and cultured in an incubator (37°C, 5% CO_2_). After 12 hours, the medium was replaced with medium containing various concentrations of tamoxifen (1, 2, 2.5, 5, 10, 20, 40, and 80 μmol/L), and the cells were cultured for an additional 48 hours. Then, 10 μL of CCK‐8 solution (Beyotime) was added to each well and the dishes were incubated for 1 hour at 37°C. The OD_450_ was measured using a microplate reader. Excel and GraphPad 6.01 were used to calculate half maximal inhibitory concentration (IC_50_) values and draw IC_50_ curves.

Five groups of 96‐well plates were prepared and inoculated with 100 μL of the cell suspension (30 000 cells/mL). The plates were cultured in an incubator (37°C, 5% CO_2_). One group was removed every 24 hours, and 10 μL of CCK‐8 solution was added to each well. The cells were incubated with CCK‐8 solution for 1 hour. Absorbance at 450 nm was measured using a microplate reader. Cell proliferation curves were drawn using GraphPad 6.01.

### 2.4 Flow cytometric analysis of apoptosis

Cells were detached with 0.25% trypsin and centrifuged at 1000 rpm for 10 minutes at 4°C. The supernatant was removed, and 1 mL of phosphate‐buffered saline (PBS) was added to each tube. The cells were washed twice, then suspended in 500 μL of binding buffer, and 10 μL of annexin V‐fluorescein isothiocyanate and 10 μL of propidium iodide (PI) were added sequentially (4A Biotech, Beijing, China). Samples were mixed in the dark. After 15 minutes, the apoptosis rate was detected by a flow cytometry (LSRFortessa, BD Biosciences).

### 2.5 Western blot analysis

Proteins were extracted from cell lysates and quantified by the Bradford assay, then separated by sodium dodecyl sulfate‐polyacrylamide gel electrophoresis and transferred to polyvinylidene difluoride membranes (EMD Millipore). Membranes were incubated with primary antibodies at 4°C overnight. The next day, after incubation with peroxidase‐coupled anti‐mouse or anti‐rabbit IgG at 37°C for 2 hours, protein levels were visualized by electrochemiluminescence. Antibodies against SIRT4 (1:40 000), STAT3 (1:2000), MYC (1:1000), and histone H3 (1:6000) were purchased from ProteinTech. Antibodies against p‐STAT3 Y705 (1:2000), CCND1 (1:1000), and glyceraldehyde 3‐phosphate dehydrogenase (GAPDH; 1:1000) were purchased from Cell Signaling Technology. Antibodies against *α*‐tubulin were purchased from Santa Cruz Biotechnology (1:1000). S3I‐201 was purchased from Selleck. Interleukin (IL)‐6 was purchased from Sino Biological.

### 2.6 Quantitative reverse transcription‐polymerase chain reaction (qRT‐PCR)

Cells in each well of a 6‐well plate were collected using 1 mL of TRIzol. Total RNA was extracted using 200 μL of trichloromethane, followed by precipitation with 500 μL of isopropanol and purification with alcohol. The PrimeScript RT Kit (TaKaRa, Dalian, China) was used to reverse transcribe RNA to cDNA, and RNA levels were quantified by real‐time quantitative PCR. The conditions were as follows: 50°C for 2 minutes, 95°C for 10 minutes, and 95°C for 15 seconds for 40 cycles, and 60°C for 1 minute. Gene expression levels were determined by normalization against *GAPDH* mRNA expression. The primer sequences were as follows:

**Table:** 

SIRT4	Forward primer	5′‐ACCCTGAGAAGGTCAAAGAGTTAC‐3′
SIRT4	Reverse primer	5′‐TTCCCCACAATCCAAGCAC‐3′
MYC	Forward primer	5′‐TGAGGAGGAACAAGAAGATG‐3′
MYC	Reverse primer	5′‐ATCCAGACTCTGACCTTTTG‐3′
CCND1	Forward primer	5′‐GCTGCGAAGTGGAAACCATC‐3′
CCND1	Reverse primer	5′‐CCTCCTTCTGCACACATTTGAA‐3′
GAPDH	Forward primer	5′‐GGAGCGAGATCCCTCCAAAAT‐3′
GAPDH	Reverse primer	5′‐GGCTGTTGTCATACTTCTCATGG‐3′

### 2.7 Immunofluorescence

Glass slides were placed in a 24‐well plate. Then, 250 μL of the cell suspension with the appropriate concentration was evenly spread on the slides. Cells were fixed with 4% paraformaldehyde (in PBS) for 20 minutes and permeabilized with 0.5% Triton X‐100 (in PBS) for 10 minutes. Goat serum was added to the slides for 1 hour at 25°C. Cells were incubated with mouse polyclonal anti‐STAT3 (1:100; ProteinTech) and placed in an incubator with high humidity at 4°C overnight. On the second day, the cells were incubated for 2 hour at room temperature with fluorescein (FITC)‐conjugated goat anti‐mouse IgG (ZSGB‐BIO, Beijing, China). DAPI (Beyotime Biotechnology, Shanghai, China) was used to stain nuclei and Antifade Mounting Medium (Beyotime Biotechnology) was used to seal the slides. Then, images were obtained using a fluorescence microscope.

### 2.8 Statistical analysis

SPSS 11.5 for Windows was used for all analyses. All data are presented as the mean ± standard deviation. Differences between groups were evaluated by Student's *t* test. *P* < .05 was considered statistically significant.

## 3 RESULTS

### 3.1 SIRT4 enhances the efficacy of tamoxifen in ER‐positive breast cancer cells

**Figure 1:**
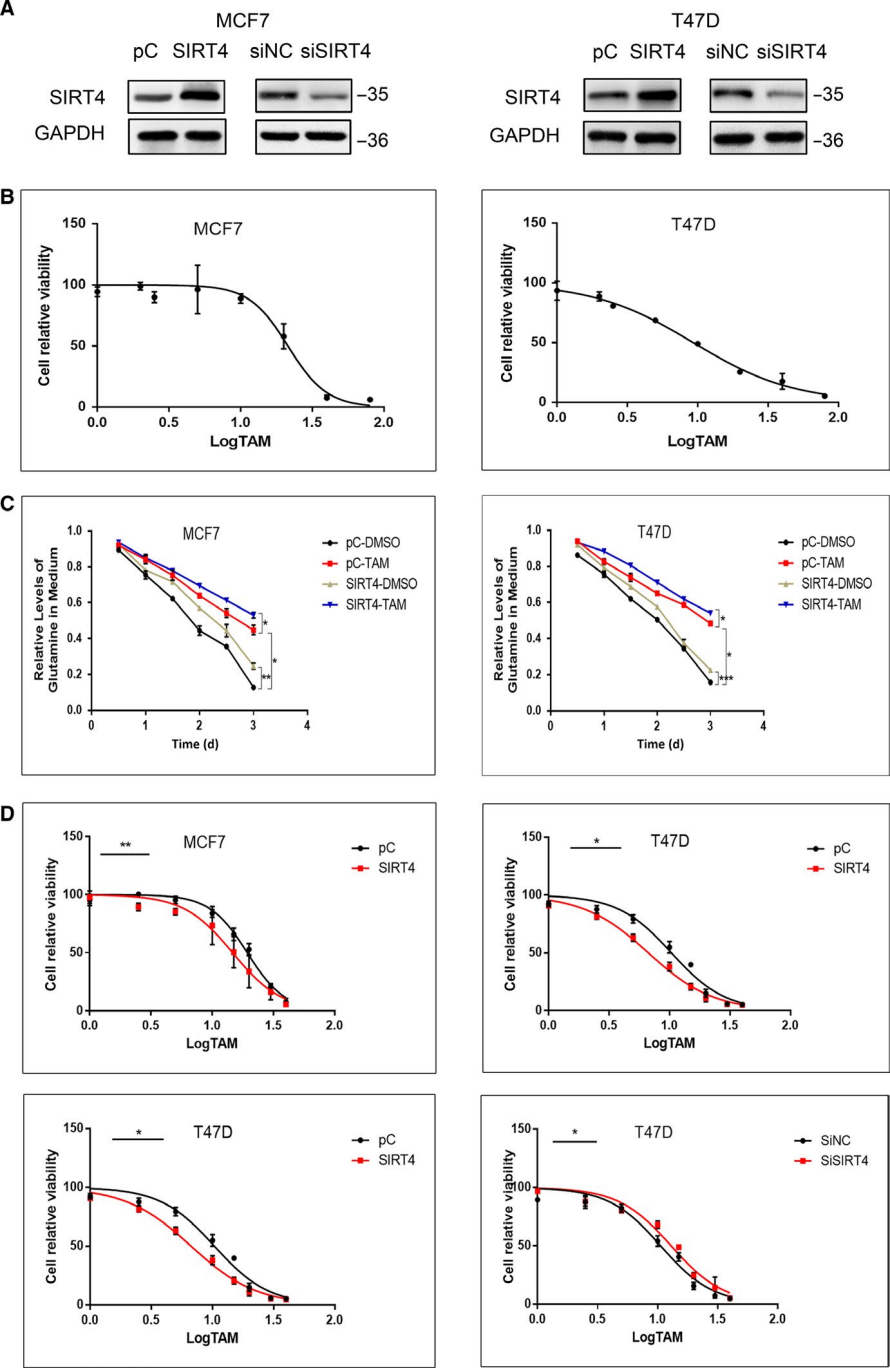
SIRT4 enhanced the efficacy of tamoxifen in MCF7 and T47D cells. (A) MCF7 and T47D cells were transfected with SIRT4 plasmids or empty vectors, and interfered with SIRT4 siRNAs or negative controls. Expression levels of SIRT4 were assessed by western blot. (B) IC50 of tamoxifen in MCF7 cell lines was 21.38 ± 2.54 and in T47D cell lines was 9.356 ± 0.78. (C) MCF7 and T47D cells were transfected with SIRT4 plasmids or empty vectors. After treated with or without tamoxifen, glutamine levels in the medium were detected by ELISA. (D) IC50 of tamoxifen in MCF7 and T47D cells were evaluated by CCK‐8 assay after SIRT4 transfection and interference. All the results were shown as mean ± SD, which were three separate experiments performed in triplicate. *P < .05, **P < .01, ***P < .001

Glutamine is an essential nutrient for cell growth and viability, and tamoxifen suppresses the proliferation of ER‐negative cells through the inhibition of glutamine uptake.^5^, ^9^ To examine if tamoxifen also reduces glutamine uptake in ER‐positive breast cancer cells and if its efficacy is affected by SIRT4, we measured glutamine levels in the medium of MCF7 and T47D cells for 3 days after SIRT4 overexpression and tamoxifen treatment. Compared with cells treated with DMSO, glutamine levels were higher in the medium of cells treated with tamoxifen. In other words, the glutamine uptake of MCF7 and T47D cells was significantly reduced after tamoxifen treatment. Thanks to the inhibitory effect of SIRT4 on glutamine metabolism, cells treated with tamoxifen after SIRT4 overexpression consumed less glutamine than cells treated with tamoxifen only (Figure 1A‐C). This suggests that SIRT4 cooperated with tamoxifen to reduce the glutamine uptake of ER‐positive breast cancer cells.

The IC_50_ can be used to report the tolerance or sensitivity of cells to drugs. To verify that SIRT4 enhances the sensitivity of ER‐positive breast cancer cells to tamoxifen, we transfected ER‐positive MCF7 and T47D breast cancer cells with plasmids containing SIRT4 or empty vectors and measured cell viability by CCK‐8 assay after treatment with different concentrations of tamoxifen. The IC_50_ value of cells overexpressing SIRT4 was lower than in the cells transfected with the empty vector. We also used SIRT4‐specific short interfering RNAs (siRNAs) to deplete SIRT4 levels in the two breast cancer cell lines, and observed that the IC_50_ increased after SIRT4 depletion (Figure 1A,D). These results confirmed that SIRT4 overexpression enhances the sensitivity of ER‐positive breast cancer cells to tamoxifen, while its absence decreases their sensitivity.

### 3.2 SIRT4 enhances tamoxifen‐induced proliferative inhibition and apoptosis in ER‐positive breast cancer cells

**Figure 2:**
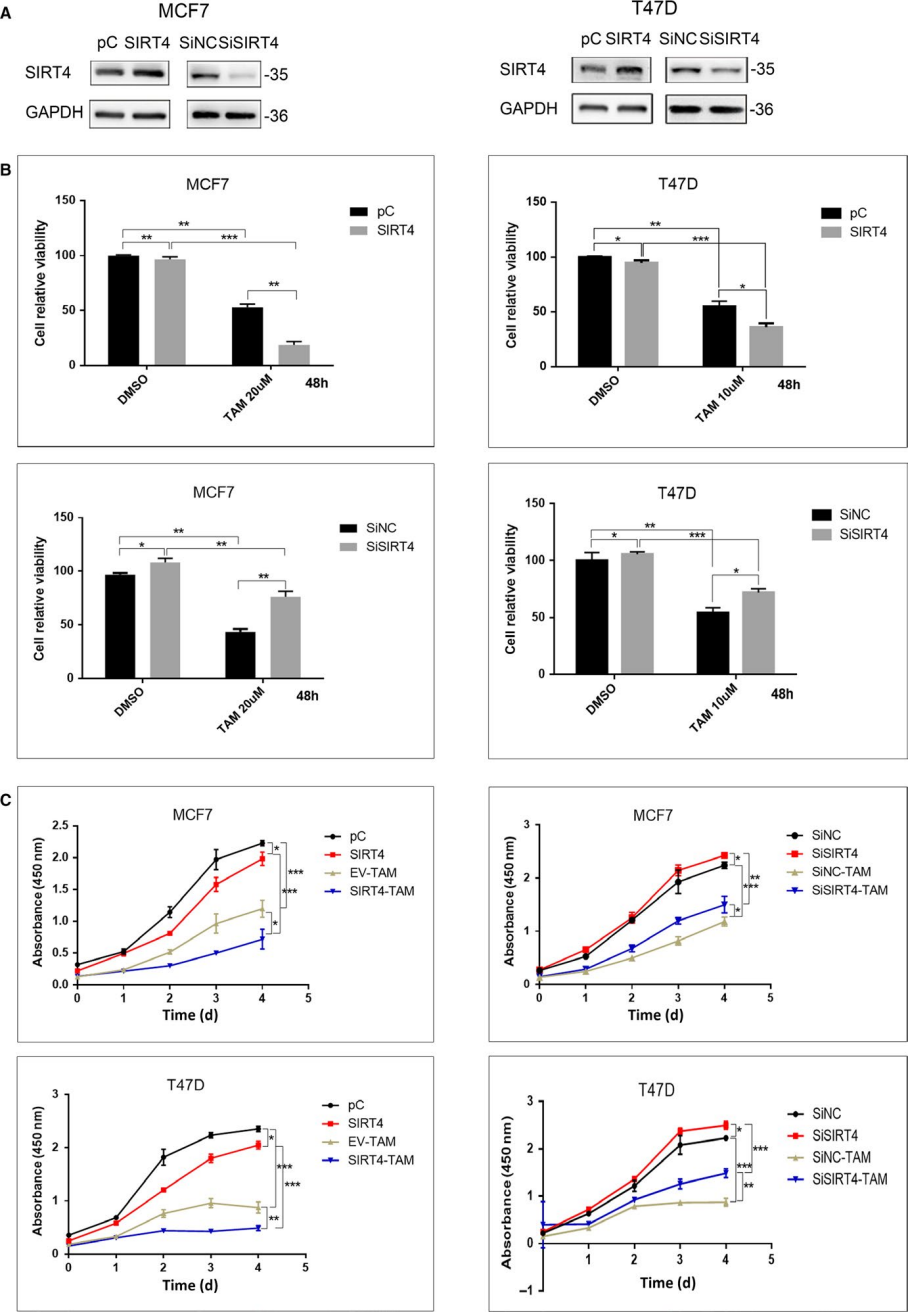
SIRT4 overexpression enhanced tamoxifen‐induced proliferation inhibition. (A) The levels of SIRT4 in MCF7 and T47D cells with SIRT4 transfection or interference were analyzed by western blot. (B) Relative activity and (C) proliferation rate of MCF7 and T47D cells with SIRT4 transfection or interference were assessed by CCK8 after exposed to the indicated dose of tamoxifen or not. All the results were shown as mean ± SD, which were three separate experiments performed in triplicate. *P < .05, **P < .01, ***P < .001

Tamoxifen kills breast cancer cells by inhibiting proliferation and promoting apoptosis. To evaluate whether the inhibitory effect of tamoxifen on proliferation was influenced by SIRT4, we compared the relative viability of breast cancer cells under a tamoxifen concentration approximately equal to the IC_50_. The breast cancer cell line MCF7 was treated with 20 μmol/L tamoxifen or DMSO for 48 hours after overexpression or depletion of SIRT4. Western Blot was used to detect the efficiency of plasmids or short interfering RNAs (Figure 2A). As shown in Figure 2B, overexpression of SIRT4 resulted in a decrease in the relative viability of the cells. Similar results were obtained when the breast cancer cell line T47D was treated with 10 μmol/L tamoxifen. We next mapped the growth curves of the two breast cancer cell lines. In both tamoxifen‐ and DMSO‐treated cells, the growth of cells with SIRT4 overexpression was significantly lower than that of cells transfected with empty vectors. Cells treated with SIRT4‐specific siRNAs had higher growth rates (Figure 2C).

**Figure 3:**
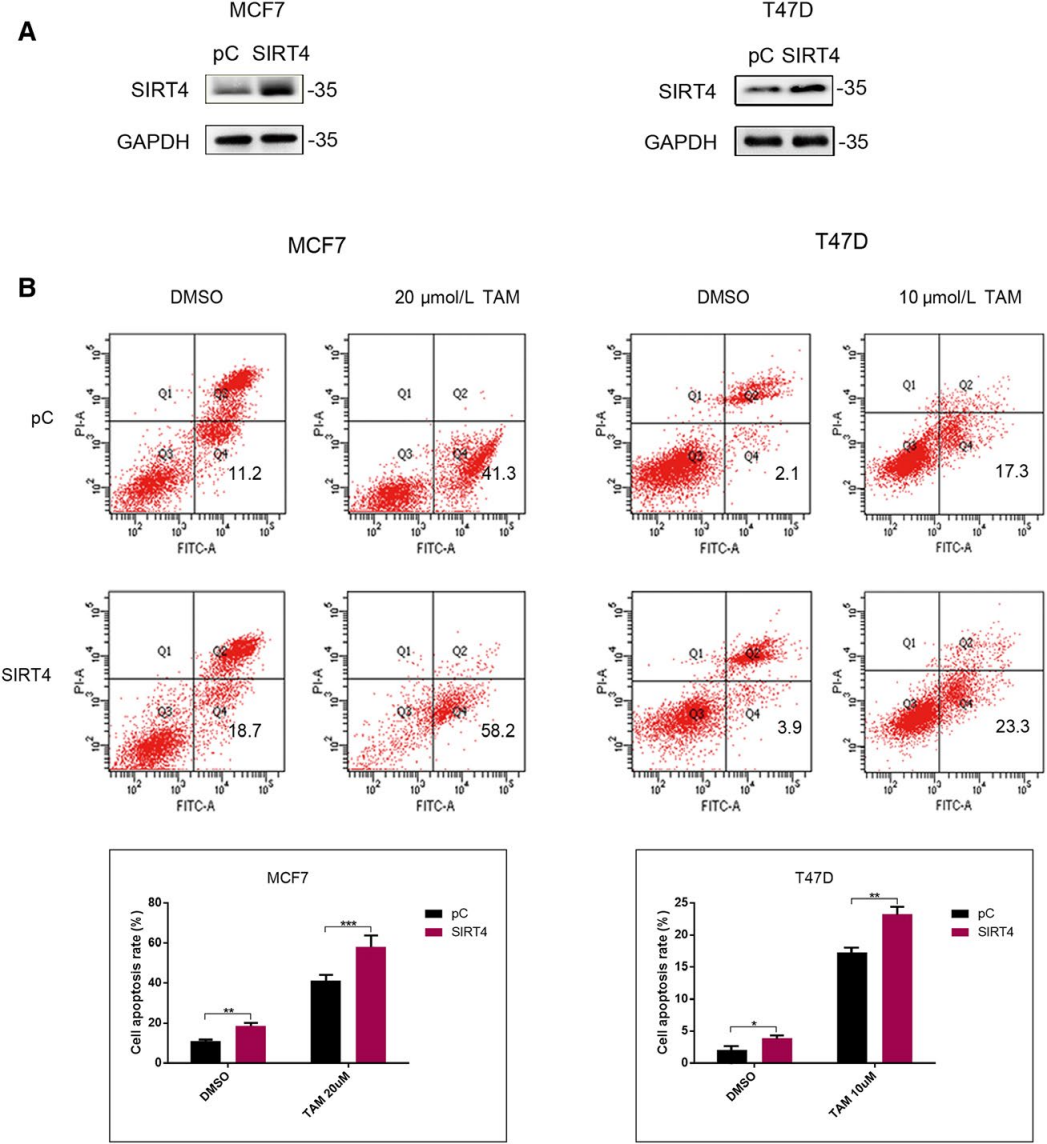
SIRT4 transfection promoted tamoxifen‐induced apoptosis. (A) MCF7 and T47D cells were transfected with SIRT4 plasmids. The transfection efficiency was detected by western blot. (B) Cell apoptosis was detected by flow cytometry analysis in both MCF7 and T47D cells treated with or without tamoxifen after SIRT4 transfection. All the results were shown as mean ± SD, which were three separate experiments performed in triplicate. *P < .05, **P < .01, ***P < .001

Since SIRT4 and tamoxifen synergistically inhibited breast cancer cell proliferation, we next evaluated whether SIRT4 also promotes tamoxifen‐induced apoptosis. Apoptosis was detected by flow cytometry after transfecting MCF7 cells with SIRT4 or an empty vector and treating them with DMSO or tamoxifen for 72 hours. Cells overexpressing SIRT4 had a higher apoptosis rate than cells transfected with empty vectors, and identical results were obtained in T47D cells. Transfection with SIRT4 promoted tamoxifen‐induced apoptosis (Figure 3).

### 3.3 SIRT4 inhibits STAT3 signaling pathway activation

As a point of convergence for many oncogenic signaling pathways, STAT3 is constitutively activated at a high frequency in a wide range of cancers and is a promising molecular target for cancer therapy. Hyperactivation of STAT3 via the constitutive phosphorylation of Y705 is common in most human cancers. Of particular note, some studies have indicated that tamoxifen‐resistant MCF‐7/TAM cells have an elevated level of tyrosine 705‐phosphorylated STAT3 compared with those of their parent cell line, MCF‐7.^10^, ^11^

**Figure 4:**
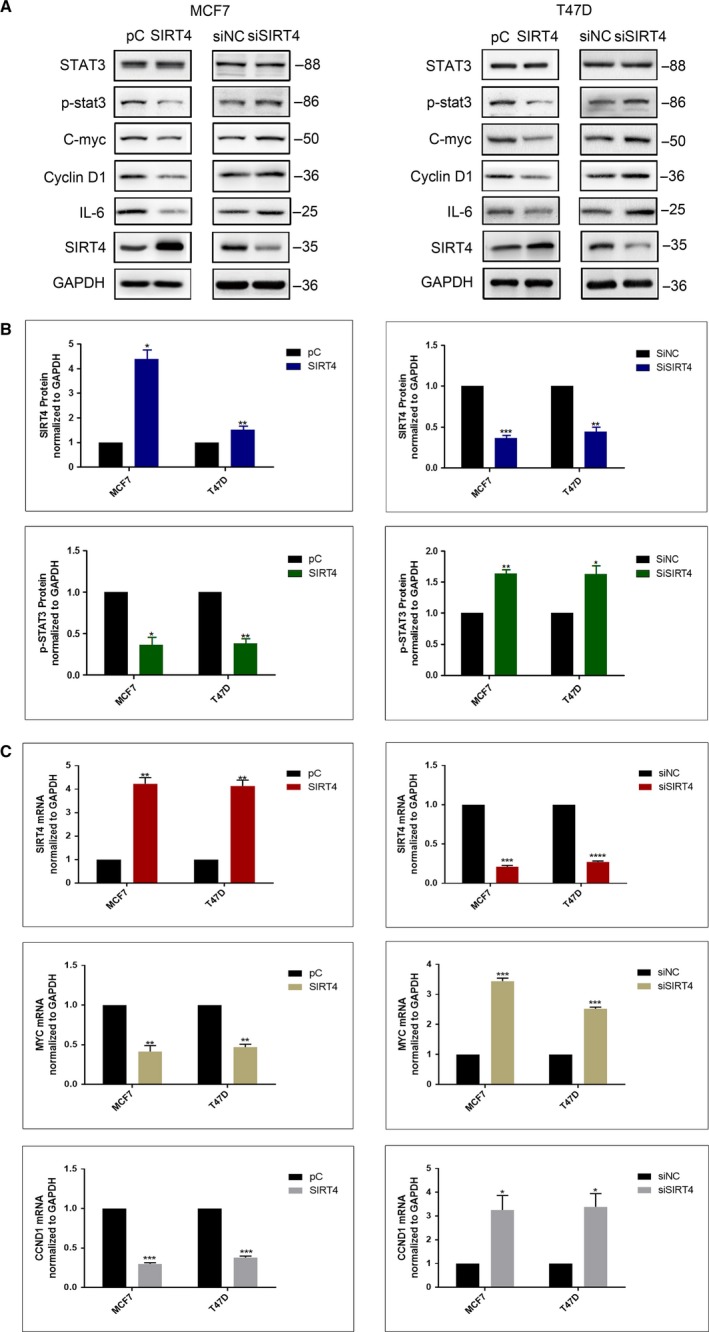
Effect of SIRT4 on indicated proteins related to STAT3 signaling pathway. (A) Protein levels of p‐STAT3, c‐myc, cyclin D1, and IL‐6 were downregulated after SIRT4 transfection or upregulated after SIRT4 interference. (B) Quantification of p‐STAT3 in MCF7 and T47D cells with SIRT4 overexpression or deletion were shown in histogram. (C) After SIRT4 transfection or interference, levels of *MYC* and *CCND1* mRNA in MCF7 and T47D cells was measured by PCR. All the results were shown as mean ± SD, which were three separate experiments performed in triplicate. *P < .05, **P < .01, ***P < .001

Therefore, we evaluated the levels of STAT3 and p‐STAT3 Y705 in MCF7 and T47D cells with SIRT4 overexpression or depletion. Western blotting showed that the expression of STAT3 was not affected by SIRT4, but STAT3 Y705 phosphorylation was downregulated with SIRT4 overexpression and upregulated after SIRT4 depletion (Figure 4A,B). These results indicate that SIRT4 inhibits STAT3 Y705 phosphorylation.

**Figure 5:**
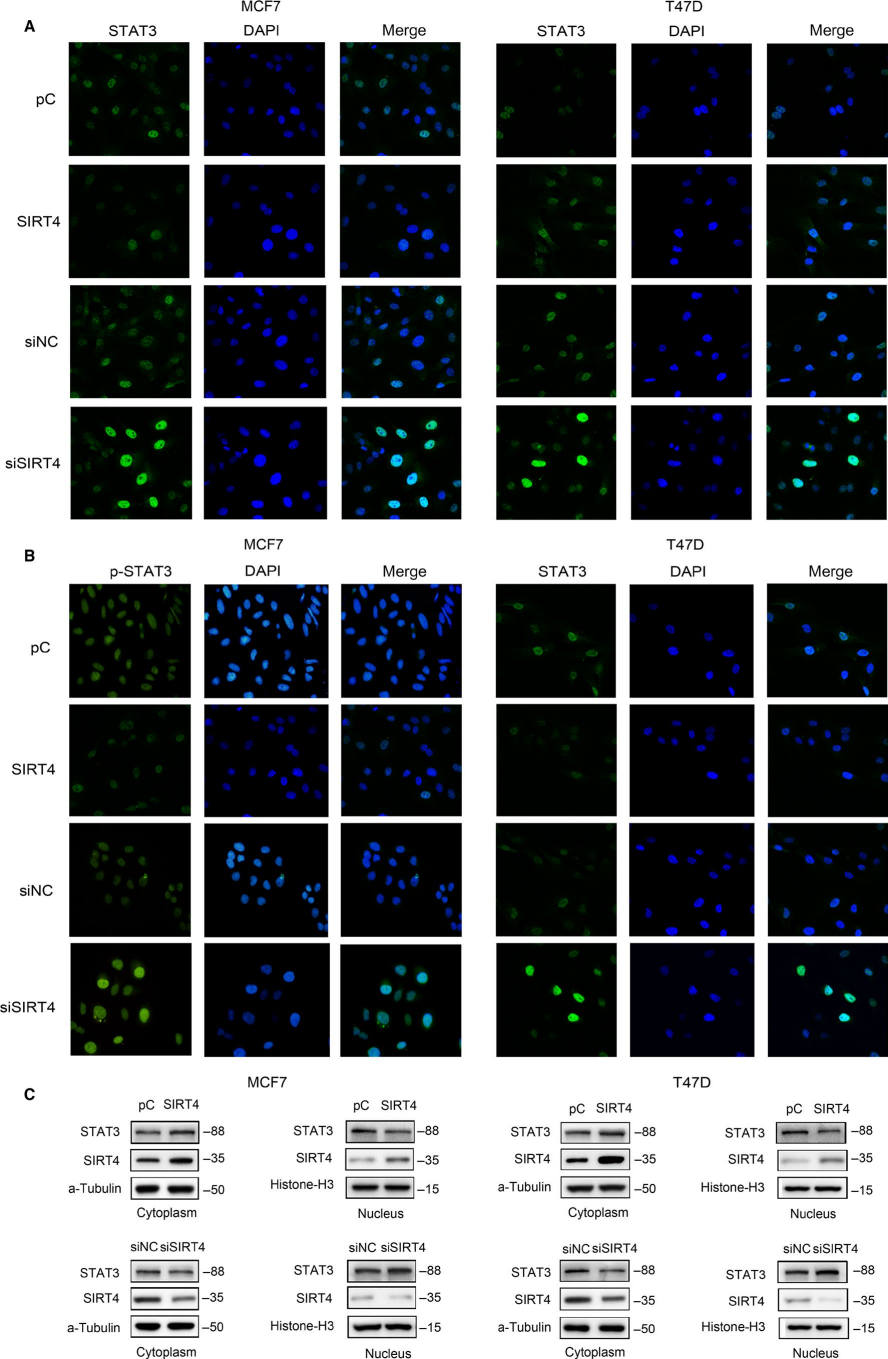
Effects of SIRT4 on STAT3 nuclear translocation. (A) Staining and localization of STAT3 and (B) p‐STAT3 in MCF7 and T47D cells after SIRT4 transfection or interference. (C) Western blot analysis for level of STAT3 expression in nucleus and cytoplasm

Once phosphorylated, STAT3 translocates to the nucleus. Therefore, immunofluorescence was used to detect the localization of STAT3 in breast cancer cells after SIRT4 overexpression or depletion. Relative to the negative controls, The STAT3 level in the nucleus was weaker with SIRT4 overexpression and stronger with SIRT4 depletion. Accordingly, the STAT3 level in the cytoplasm was stronger with SIRT4 overexpression and weaker with SIRT4 depletion (Figure 5A). Immunofluorescence of p‐STAT3 Y705 was also weaker with SIRT4 overexpression (Figure 5B). To verify this finding, the nucleus and cytoplasm of cells were separated by the nucleoplasmic protein isolation technique after transfection with SIRT4 plasmids. Western blotting showed that, compared with the negative control cells, STAT3 expression was weaker in the nucleus and stronger in the cytoplasm following SIRT4 overexpression in both breast cancer cell lines. After SIRT4 depletion, the opposite result was obtained (Figure 5C). These results are consistent with those obtained by immunofluorescence and confirm our hypothesis that SIRT4 inhibits STAT3 phosphorylation and nuclear translocation, suppressing the STAT3 signaling pathway.

### 3.4 SIRT4 inhibits the transcription and translation of MYC and CNDD1

As targets of the STAT3 signaling pathway, *MYC* and *CCND1* gene amplifications confer tamoxifen resistance in ER‐positive breast cancer.^7^, ^8^, ^11^, ^12^ Accordingly, we evaluated whether SIRT4 affects their expression. After up‐ and downregulation of SIRT4 in the two cell lines, we detected MYC and CCND1 expression by western blotting. MYC and CCND1 levels decreased after SIRT4 overexpression, and increased after SIRT4 depletion (Figure 4A).

We also evaluated whether SIRT4 regulates expression of MYC and CCND1 at the transcriptional level by quantitative reverse transcription‐polymerase chain reaction. As predicted, SIRT4 overexpression resulted in decreased *MYC* and *CCND1* transcription, while its depletion resulted in increased transcription of these genes (Figure 4C). These results are in agreement with our hypothesis that SIRT4 enhances the sensitivity of ER‐positive breast cancer cells to tamoxifen by decreasing the transcription and translation of the oncogenes *MYC* and *CCND1*.

### 3.5 SIRT4 enhances the sensitivity of breast cancer cells to tamoxifen by inhibiting the STAT3 signaling pathway

**Figure 6:**
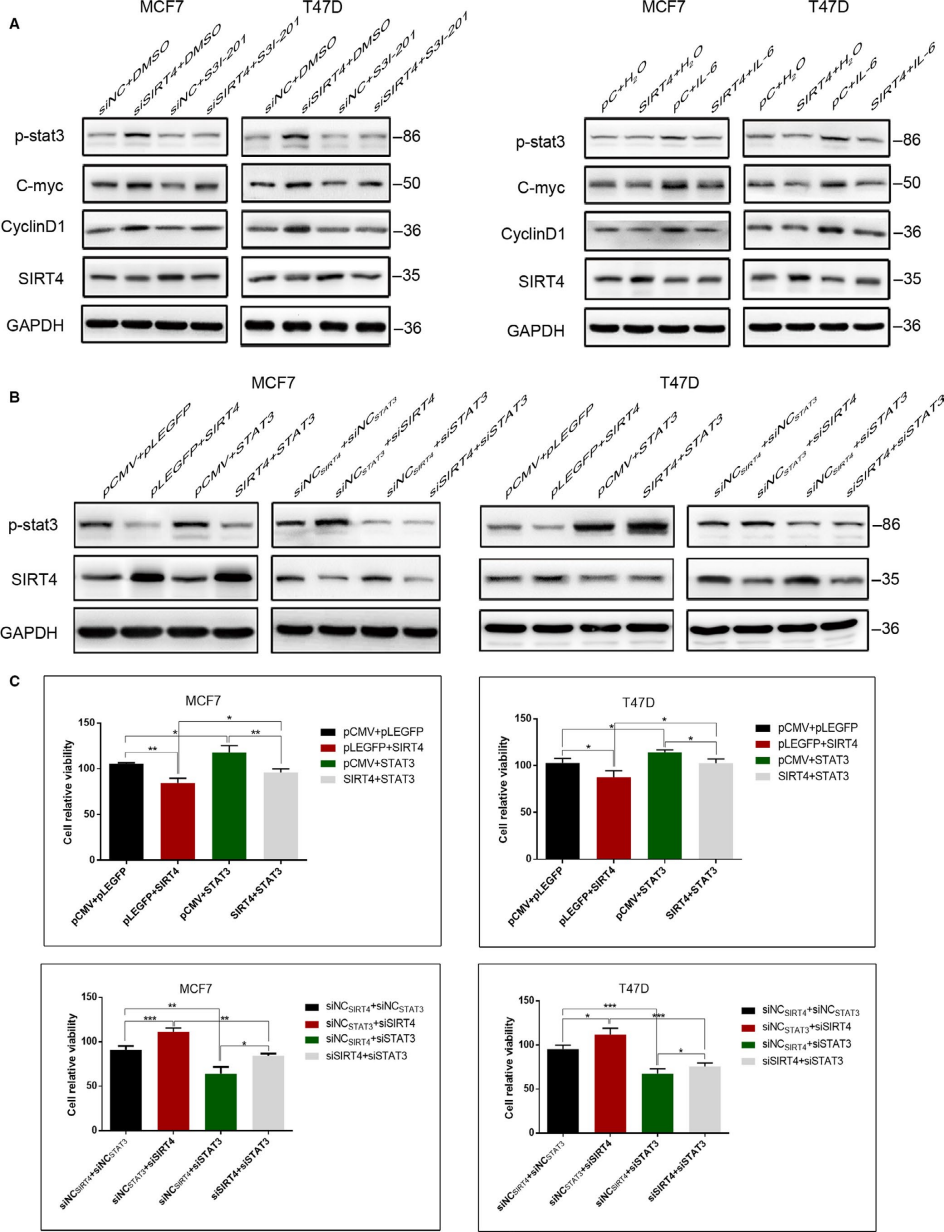
Effects of SIRT4 on sensitivity of MCF7 and T47D cells to tamoxifen were reversed by STAT3. (A) MCF7 and T47D cells were treated with IL‐6 or S3I‐201 after SIRT4 transfection or interference. Expression levels of tamoxifen resistance‐related proteins c‐myc and cyclin D1 were determined by western blot. (B) Levels of SIRT4 and p‐STAT3 were detected by western blot in MCF7 and T47D cells transfected or interfered with SIRT4 and STAT3 together, or replaced by respective empty vectors. (C) Relative activity of MCF7 and T47D cells was analyzed by CCK‐8 with tamoxifen treatment after cotransfection. All the results were shown as mean ± SD, which were three separate experiments performed in triplicate. *P < .05, **P < .01, ***P < .001

IL‐6 is an activator of the STAT3 pathway.^12^ Breast cancer cells were treated with or without IL‐6 after transfection with SIRT4 plasmids or empty vectors. We detected p‐STAT3 Y705, MYC, and CCND1 levels by western blotting. Relative to those in cells transfected with empty plasmids, the levels of p‐STAT3 Y705, MYC, and CCND1 were lower with SIRT4 overexpression. In cells treated with IL‐6 in the presence of SIRT4 overexpression, the levels of these proteins were higher than in untreated cells transfected with SIRT4. These results suggested that STAT3 activation can offset the decreases in MYC and CCND1 caused by SIRT4 overexpression. As an inhibitor of the STAT3 pathway, S3I‐201 can inhibit the persistent activation of STAT3 and the expression of STAT3 target genes, such as *MYC, CCND1,* and MCL1 apoptosis regulator, BCL2 family member.^6^, ^12^, ^13^ Therefore, control cells and cells depleted of SIRT4 for 48 hours were either treated with S3I‐201 or left untreated, and western blotting was used to quantify the levels of p‐STAT3 Y705, MYC, and CCND1. As shown above, their levels were higher with SIRT4 depletion. However, S3I‐201 treatment was able to offset these effects in SIRT4‐depleted cells, suggesting that SIRT4 depletion cannot affect MYC and CCND1 expression after STAT3 pathway inhibition. In summary, SIRT4 depresses the expression of tamoxifen resistance‐related proteins through STAT3, enhancing the cell's sensitivity to tamoxifen (Figure 6A). We next sought to determine whether STAT3 could reverse the effects of SIRT4 on the response of breast cancer cells to tamoxifen. Cells were transfected with both SIRT4 and STAT3 plasmids, with empty plasmids transfected into negative control cells, and either SIRT4 or STAT3 plasmids transfected into positive control cells (Figure 6B). After transfection, cells were treated with tamoxifen for 48 hours and their viability was measured. Relative to the negative controls, the viability of cells with SIRT4 overexpression decreased, and the viability of cells with STAT3 overexpression increased. The viability of cells cotransfected with SIRT4 and STAT3 plasmids was higher than that of cells transfected with SIRT4 plasmids or empty vectors. We repeated the analyses using depletion instead of overexpression. Cell viability after SIRT4 depletion group was lower than in the negative controls, while it was higher with STAT3 depletion. Cells with codepletion of SIRT4 and STAT3 had reduced proliferation rates compared with cells transfected with SIRT4 siRNAs and negative control siRNAs (Figure 6C). Taken together, these results suggest that SIRT4 affects the sensitivity of ER‐positive breast cancer cells to tamoxifen via the STAT3 pathway.

### 3.6 SIRT4 inhibits STAT3 pathway activation by downregulating IL‐6

STAT3 signaling is stimulated by cytokines such as IL‐6. To evaluate whether SIRT4 inhibits STAT3 pathway activation by downregulating IL‐6, we compared the levels of IL‐6 between cells transfected with SIRT4 plasmids and empty plasmids by western blotting. As expected, SIRT4 overexpression resulted in significantly lower expression of IL‐6 compared to that in control cells, while its expression after SIRT4 depletion was obviously higher (Figure 4A). In addition, the level of p‐STAT3 Y705 in breast cancer cells with SIRT4 transfection and IL‐6 treatment was higher than that in cells with SIRT4 transfection only. These results suggest that the inhibition of the STAT3 signaling pathway by SIRT4 is reversed by IL‐6 (Figure 6A), indicating that SIRT4 suppresses STAT3 activation by decreasing IL‐6.

## 4 DISCUSSION

SIRT4 was first identified as a mitochondrial protein that controls energy metabolism, and early studies focused on its role in metabolic diseases and obesity.^14^, ^15^ Years later, researchers at Harvard Medical School and the U. S. National Institutes of Health discovered that SIRT4 has tumor‐suppressive activity and regulates the cellular metabolic response to DNA damage by inhibiting mitochondrial glutamine metabolism.^16^, ^17^ *SIRT4* is downregulated in a variety of human malignant tumors, including gastric cancer, colorectal cancer, lung cancer, and liver cancer, confirming its function as a tumor suppressor.^18^, ^19^, ^20^, ^21^, ^22^, ^23^ Thus, the link between SIRT4 and cancer came to light, and studies of the locus entered a new stage. In an investigation of the precise roles of sirtuin genes, high‐throughput real‐time PCR analysis showed that *SIRT4* expression was significantly lower in breast cancer tissues than in adjacent breast tissues.^24^ Another study showed that C‐terminal‐binding protein promotes growth and represses apoptosis in the breast cancer cell lines MCF‐7 and MDA‐MB‐231 by downregulating SIRT4.^25^, ^26^ These findings indicate that *SIRT4* acts as a tumor suppressor gene, inhibiting cell growth and promoting apoptosis in breast cancer.

Glutamine plays a critical role in cellular growth in multiple cancers. Our study demonstrates that tamoxifen can inhibit glutamine uptake in MCF‐7 and T47D cells, and that SIRT4 enhances this effect. To examine whether SIRT4 augments the effects of tamoxifen on breast cancer, overexpressed and depleted the protein in ER‐positive MCF7 and T47D breast cancer cells. As predicted, SIRT4 overexpression resulted in reduced an IC_50_ value for tamoxifen, decreased relative viability and proliferation, and increased apoptosis, while depletion had the opposite effects. Accordingly, our results indicate that SIRT4 enhances the sensitivity of ER‐positive breast cancer to tamoxifen. This is consistent with a previous study showing that SIRT4 knockout decreases chemosensitivity to fluorouracil in colorectal cancer cells.^20^

STAT3 is activated in breast cancer, and Y705 phosphorylation is elevated in MCF7 cells that are resistant to tamoxifen.^11^ Glutamine regulates cancer cell invasiveness via STAT3, and its deprivation significantly decreases the levels of STAT3 phosphorylation at Y705 in highly invasive ovarian cancer cells.^27^ Xia et al demonstrated that SIRT4 can induce SMCC7721 liver cancer cell aging by suppressing JAK2/STAT3 signaling, consistent with our observations in breast cancer.^28^ However, the molecular mechanisms controlling this were not discussed. In this study, we provide the first evidence of a link between SIRT4 and the STAT3 signal pathway. Our analysis of SIRT4 and p‐STAT3 levels in MCF‐7 and T47D cells showed that SIRT4 is negatively correlated with p‐STAT3 Y705. STAT3 activation counteracted the effects of SIRT4, not only on the expression of tamoxifen resistance‐related genes but also on cell proliferation. Similar to results obtained in glucose‐stimulated podocytes, the release of IL‐6 was reduced in ER‐positive breast cancer cells when SIRT4 was overexpressed,^29^ and after IL‐6 treatment, SIRT4‐induced inhibition of STAT3 was reversed. This indicates that SIRT4 enhances the sensitivity of ER‐positive breast cancer to tamoxifen by inhibiting the IL‐6/STAT3 pathway. In colon carcinoma cells, glutamine deprivation essentially shuts down translation of MYC and re‐addition of glutamine restores translation.^30^ In our study, SIRT4 overexpression decreased *MYC* expression significantly, as well as *CCND1*, another marker of tamoxifen resistance. *MYC* and *CCND1* amplification reduce the sensitivity of ER‐positive breast cancer to tamoxifen, but SIRT4 enhances this sensitivity by suppressing targets of the STAT3 pathway.

This study demonstrates that SIRT4 enhances the sensitivity of breast cancer to tamoxifen via STAT3 pathway inhibition due to decreased STAT3 Y705 phosphorylation. However, the mechanisms behind cancer occurrence and drug resistance are complicated, and the relationships between the factors involved are diverse. For example, in addition to entering the nucleus to regulate target gene transcription, STAT3 can also enter the mitochondria to regulate ATP synthesis. This process depends on another STAT3 phosphorylation site at serine 727.^31^ SIRT4 can regulate ATP homeostasis.^32^ Accordingly, whether SIRT4 affects the efficacy of tamoxifen by regulating STAT3 S727 phosphorylation is worth investigating. It should be noted that this study was performed in two ER‐positive breast cancer cell lines, and the role of SIRT4 in the sensitivity of ER‐negative breast cancer to tamoxifen remains to be verified. The results of the in vitro experiments reported here should also be confirmed in vivo in future studies.

As a gene related to energy metabolism, *SIRT4* acts as a tumor suppressor in many cancers. This study focused on the role of SIRT4 in the tamoxifen sensitivity of breast cancer and demonstrated that SIRT4 blocks the transcription and translation of *MYC* and *CCND1* in ER‐positive breast cancer, through the STAT3 pathway. Accordingly, our findings suggest that SIRT4 plays an important role in the sensitivity of ER‐positive breast cancer to tamoxifen and should be further studied to address the issue of drug resistance to endocrine therapies for breast cancer.

## CONFLICT OF INTEREST

The authors have no conflict of interest.

## Data Availability

The raw data supporting the conclusions of this manuscript will be made available by the authors, without undue reservation, to any qualified researcher.
